# Severe lupus pneumonitis: A case of life‐saving multimodal therapy with rituximab and corticosteroids

**DOI:** 10.1002/rcr2.1426

**Published:** 2024-07-03

**Authors:** Amirhossein Aarabi, Stella McGinn

**Affiliations:** ^1^ Renal Department Royal North Shore Hospital Sydney New South Wales Australia

**Keywords:** autoimmune disease, inflammation, lupus pneumonitis, rare lung diseases

## Abstract

Severe lupus pneumonitis is a rare and life‐threatening complication of systemic lupus erythematosus (SLE), characterized by its rapid progression and high mortality rate. This case report describes the clinical trajectory and therapeutic management of a young Aboriginal female with established lupus nephritis who developed severe lupus pneumonitis. Despite her stable renal condition under long‐term immunosuppressive treatment, she experienced acute respiratory distress, leading to her admission to the intensive care unit and subsequent mechanical ventilation. The diagnostic process was complicated by the difficulty in obtaining tissue biopsies, necessitating reliance on clinical judgement and radiological evidence to formulate a diagnosis. The patient was treated with pulsed intravenous methylprednisolone followed by rituximab infusions, resulting in significant clinical and radiological improvement. This case highlights the importance of early and intensive immunosuppressive therapy in the management of severe lupus pneumonitis and underscores the utility of a multidisciplinary approach in overcoming diagnostic ambiguities. Furthermore, it contributes to the growing body of evidence supporting the efficacy of rituximab in severe lupus pneumonitis cases, offering insights into potential therapeutic avenues when conventional management strategies are inadequate or unsuitable.

## Graphical abstract

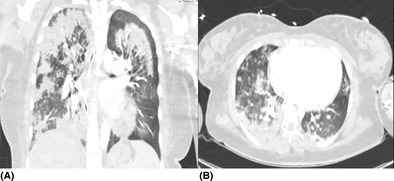

## INTRODUCTION

Systemic lupus erythematosus (SLE) is a prototypical autoimmune disorder characterized by diverse clinical manifestations that reflect its systemic nature, potentially affecting any organ system. The disease typically follows a course of intermittent flares and remissions. Of particular concern are its pulmonary manifestations, especially lupus pneumonitis, which is marked by rapid progression and is associated with significant morbidity and mortality.^1^

Lupus pneumonitis, a rare but severe pulmonary complication of systemic lupus erythematosus (SLE), requires prompt identification and aggressive management to prevent its potentially lethal progression. This condition is characterized by acute respiratory distress and inflammatory infiltrates within the pulmonary parenchyma. The rarity of lupus pneumonitis presents significant diagnostic and therapeutic challenges, further exacerbated by the limited evidence available to guide management strategies.^2^

The management of lupus pneumonitis has traditionally relied on high‐dose corticosteroids as the cornerstone of therapy, supplemented by immunosuppressants such as cyclophosphamide in cases that are refractory to initial treatment. More recently, biological agents like rituximab have been incorporated into treatment regimens for their potential benefits in resistant cases. The scarcity of randomized controlled trials and the inherent heterogeneity of the disease pose significant challenges in developing standardized treatment protocols.^3^

## CASE REPORT

A 24‐year‐old Aboriginal female with a documented history of Class IV lupus nephritis, under long‐term management with hydroxychloroquine (200 mg twice daily), mycophenolate mofetil (360 mg twice daily), and a maintenance dose of prednisone (5 mg daily), presented with fever, productive cough, and worsening shortness of breath over 3 days. The patient's medical history was notable for rheumatic heart disease and autoimmune haemolytic anaemia. Despite her complex autoimmune profile, including triple positive lupus antiphospholipid antibodies, she had no recorded incidents of thrombotic events. Upon presentation, her clinical assessment revealed tachypnoea (respiratory rate of 28 breaths per minute), fever (38.6°C), and oxygen saturation of 88% on room air, which improved to 92% with supplemental oxygen. Physical examination identified bibasilar crepitant crackles on lung auscultation and a pronounced pansystolic murmur, in line with her known history of rheumatic valvular disease. Initial laboratory investigations as demonstrated in Table 1 indicated anaemia (haemoglobin 63 g/L), leucocytosis (white cell count 6.8 × 10^9^/L), and thrombocytopenia (platelet count 91 × 10^9^/L). Renal function was mildly impaired, with a creatinine level of 146 μmol/L, aligning closely with her baseline. Autoimmune serology revealed elevated ANA and dsDNA levels (>667 IU/mL), with concomitantly low complement components (C3 at 0.32 g/L and C4 at 0.04 g/L), indicative of active systemic autoimmune activity.

**TABLE 1:** Investigations findings.

Parameter	Findings
Laboratory investigations	Anaemia (Haemoglobin: 63 g/L). Leucocytosis (White cell count: 6.8 × 10^9^/L). Thrombocytopenia (Platelet count: 91 × 10^9^/L). Mildly impaired kidney function (Creatinine: 146 μmol/L). Elevated ANA and dsDNA levels (667 IU/mL). Low complement components (C3: 0.32 g/L, C4: 0.04 g/L).
Imaging studies	Chest x‐ray and high‐resolution CT: Bilateral pulmonary infiltrates, predominance in the upper lobes. CT pulmonary angiogram: No pulmonary emboli, extensive ground‐glass opacities, and consolidation areas.
Bronchoscopy with bronchoalveolar lavage	Negative for infectious pathogens and malignancy. No hemosiderin‐laden macrophages.

**FIGURE 1:**
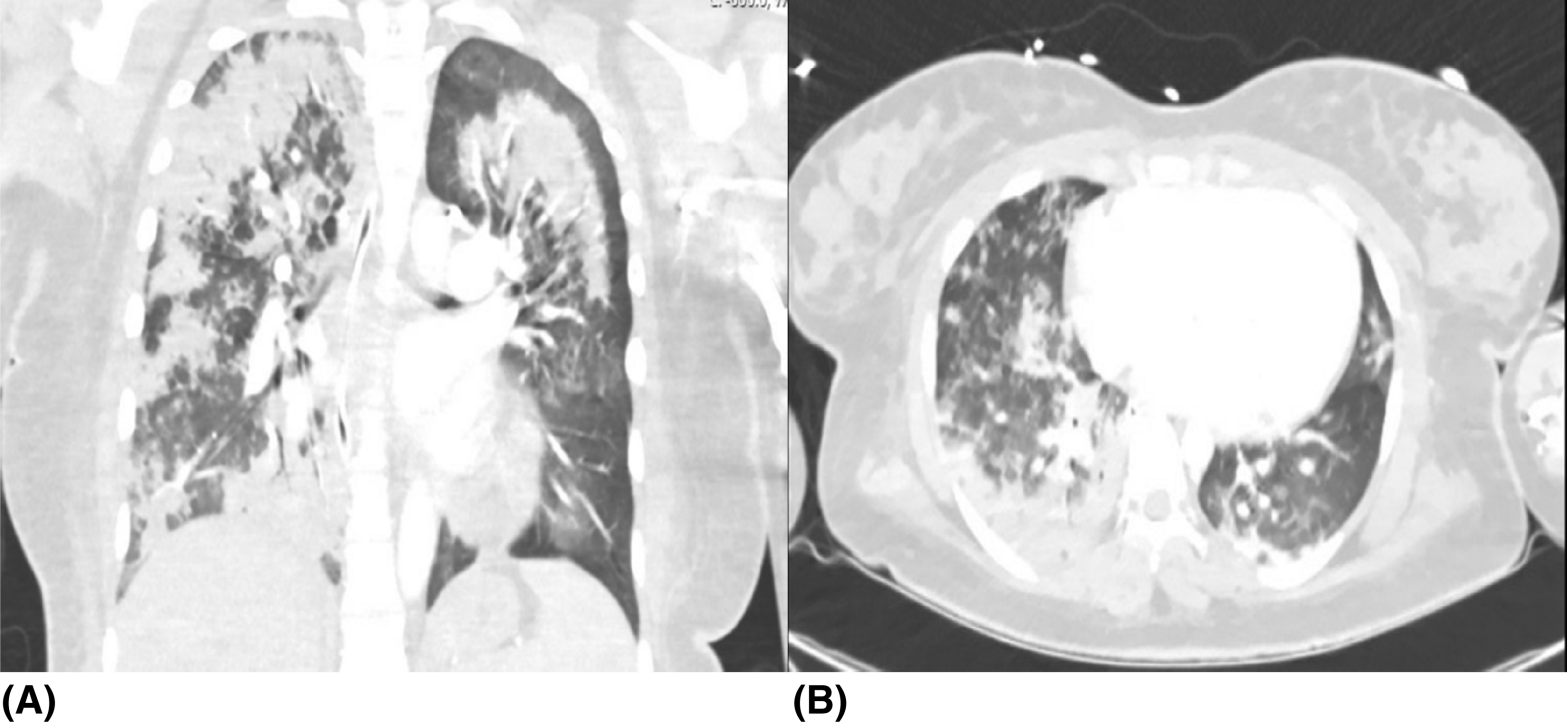
Extensive bilateral consolidations with adjacent ground glass changes prior to treatment.

Imaging studies as shown in Figure 1, including high‐resolution CT, exhibited bilateral pulmonary infiltrates with a predominance in the upper lobes, raising differential diagnoses of atypical pneumonia, acute interstitial pneumonitis, or a lupus‐related pulmonary complication. A CT pulmonary angiogram was performed to exclude thromboembolic disease, confirming the absence of pulmonary emboli but revealing extensive ground‐glass opacities and consolidation areas. Bronchoscopy with bronchoalveolar lavage yielded negative results for infectious pathogens and malignancy, and was devoid of hemosiderin‐laden macrophages, diminishing the likelihood of alveolar haemorrhage.

Given the constellation of clinical, serological, and radiographic findings, and the exclusion of infectious causes, acute lupus pneumonitis was deemed the most probable diagnosis. The patient's respiratory status deteriorated necessitating ICU admission and mechanical ventilation that made performing lung biopsy very challenging. The pressing clinical scenario prompted the initiation of pulsed intravenous methylprednisolone (1 g daily for 3 days), followed by a tapered regimen of oral prednisone. In addition, Rituximab (1 g administered 2 weeks apart) was added to the regimen, reflecting the severity of the presentation and the partial response to conventional immunosuppressive therapies in her past. Mycophenolate mofetil was discontinued during this period to mitigate infection risk and due to its potential inefficacy in rapidly progressive lupus pneumonitis.

**FIGURE 2:**
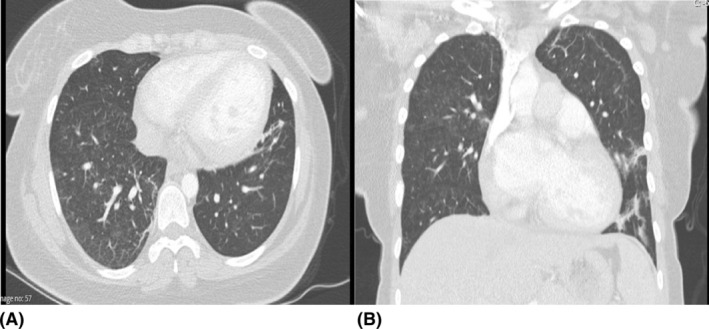
Repeat CT scan 3 weeks post commencement of therapy shows near complete resolution of diffuse consolidations.

The therapeutic response was monitored through clinical observation, serial lung imaging, and laboratory markers of lupus activity. Remarkable clinical and radiological improvement was noted, with the patient being successfully weaned off mechanical ventilation. Follow‐up chest imaging as shown in Figure 2, demonstrated substantial resolution of the infiltrates, and serological tests reflected a downtrend in dsDNA titres with a modest rebound in complement levels. This clinical course corroborated the diagnosis of lupus pneumonitis and validated the efficacy of the treatment strategy.

Upon stabilization, the patient was discharged with a comprehensive plan involving a tapered prednisone dose and continued hydroxychloroquine therapy, alongside regular outpatient rheumatology and pulmonary follow‐up appointments to monitor for potential relapses and manage her chronic lupus nephritis.^4^

## DISCUSSION

This case report describes a severe case of lupus pneumonitis in a patient with established lupus nephritis, emphasizing the intricate interplay of clinical manifestations, diagnostic hurdles, and the critical need for tailored therapeutic strategies. As a relatively rare but serious complication of systemic lupus erythematosus (SLE), lupus pneumonitis necessitates prompt and effective intervention to prevent its potentially lethal outcomes.

The diagnosis of lupus pneumonitis presents significant challenges due to its nonspecific clinical manifestations that often mimic other pulmonary disorders. Clinical assessment, reinforced by serological and radiological evaluations, remains essential, especially when obtaining histopathological confirmation is impractical. This diagnostic approach aligns with the current literature and supports a thorough evaluative strategy to effectively address the inherent complexities of diagnosing lupus pneumonitis.^3^

The pathophysiology of lupus pneumonitis is characterized by systemic immune dysregulation typical of SLE, involving autoantibody production, immune complex deposition, and complement activation. These pathogenic mechanisms suggest a substantive rationale for utilizing B‐cell depletive therapies such as rituximab. Rituximab, which specifically targets CD20+ B cells, plays a crucial role in moderating the autoimmune cascade by reducing B‐cell mediated pathology. This effect is pivotal in managing autoimmune‐driven pulmonary damage, which is central to lupus pneumonitis.^5^ In a landmark study by Jones et al., rituximab was shown to significantly reduce pulmonary symptoms and improve lung function metrics in patients with lupus pneumonitis, presenting a stronger clinical improvement profile compared to those managed with cyclophosphamide alone.^6^

Beyond rituximab, other therapeutic avenues for lupus pneumonitis include high‐dose corticosteroids, which are traditionally the first line of treatment due to their potent anti‐inflammatory effects. However, the chronic use of corticosteroids is often limited by significant side effects, including an increased risk of infections and bone density loss. This limitation underscores the importance of supplementing or replacing corticosteroids with other immunosuppressants in certain clinical scenarios.

In addition to cyclophosphamide, mycophenolate mofetil has also been explored as an alternative, particularly for patients who exhibit poor tolerance to cyclophosphamide or who are at high risk for its toxic effects. Comparative studies, such as those by Chen et al, have indicated that mycophenolate mofetil may offer comparable efficacy to cyclophosphamide with a more favourable safety profile, although evidence specific to lupus pneumonitis remains relatively sparse.^7^

The efficacy of plasma exchange as a therapeutic intervention has been investigated, particularly in cases exhibiting severe manifestations of SLE with refractory response to conventional therapies. Smith et al., highlighted that while plasma exchange can be effective in removing pathogenic autoantibodies and immune complexes, its benefits are often transient and should be considered as an adjunct to pharmacological management rather than a standalone treatment.^8^

This growing body of evidence, combined with the clinical insights from the present case, underscores the necessity for a refined approach to managing lupus pneumonitis. It advocates for a tailored treatment plan that judiciously balances the effectiveness of available therapies against their potential side effects.

In conclusion, this case of severe lupus pneumonitis effectively managed with rituximab and corticosteroids, highlights the critical severity and urgent therapeutic needs associated with this manifestation of systemic lupus erythematosus (SLE). It emphasizes the necessity for prompt and aggressive therapeutic interventions and supports the emerging evidence of rituximab's efficacy. Furthermore, this case contributes valuable insights to the existing body of literature on lupus pneumonitis and sets a precedent for future clinical and research endeavours.^9^

## CONFLICT OF INTEREST STATEMENT

None declared.

## ETHICS STATEMENT

All procedures performed in studies involving human participants were in accordance with the ethical standards of the institutional and/or national research committee and with the 1964 Helsinki declaration and its later amendments or comparable ethical standards. Informed consent was obtained from all individual participants included in the study. The authors declare that appropriate written informed consent was obtained for the publication of this manuscript and accompanying images.

## Data Availability

The data that support the findings of this study are available from the corresponding author upon reasonable request.

## References

[1] Ginzler EM , Dooley MA . Lupus nephritis: pathology, clinical course, and treatment. Am J Kidney Dis. 2010;55(1):335–358.19926370

[2] He Y , Sawalha AH . Drug‐induced lupus erythematosus: an update on drugs and mechanisms. Curr Opin Rheumatol. 2020;32(5):494–502. 10.1097/BOR.0000000000000522 PMC7299070 29870500

[3] Keane MP , Lynch JP . Pleuropulmonary manifestations of systemic lupus erythematosus. Thorax. 2000;55(2):159–166. 10639536 10.1136/thorax.55.2.159 PMC1745678

[4] Alshaiki F , Obaid E , Almuallim A , Taha R , El‐Haddad H , Almoallim H . Outcomes of rituximab therapy in refractory lupus: a meta‐analysis. Eur J Rheumatol. 2018;5(2):118–126. 10.5152/eurjrheum.2018.17096 30185361 PMC6072690

[5] Tselios K , Gladman DD , Touma Z , Su J , Anderson N , Urowitz MB . Disease course patterns in systemic lupus erythematosus. Lupus. 2019;28(1):114–122. 10.1177/0961203318817132 30526328

[6] Jones RB , Cohen Tervaert JW , Hauser T , et al. Rituximab versus cyclophosphamide in ANCA‐associated renal vasculitis. N Engl J Med. 2010;363(3):211–220. 10.1056/NEJMoa0909169 20647198

[7] Chen W , Hong SH , Jenks SA , et al. Distinct transcriptomes and autocrine cytokines underpin maturation and survival of antibody‐secreting cells in systemic lupus erythematosus. Nat Commun. 2024;15:1899. 10.1038/s41467-024-46053-w 38429276 PMC10907730

[8] Smith KGC , Jones RB , Burns SM , Jayne DRW . Long‐term comparison of rituximab treatment for refractory systemic lupus erythematosus and vasculitis: remission, relapse, and re‐treatment. Arthritis Rheum. 2006;54:2970–2982. 10.1002/art.22046 16947528

[9] Fanouriakis A , Kostopoulou M , Alunno A , et al. 2019 update of the EULAR recommendations for the management of systemic lupus erythematosus. Ann Rheum Dis. 2019;78(6):736–745. 30926722 10.1136/annrheumdis-2019-215089

